# Whole genome sequencing of drug resistance *Mycobacterium tuberculosis* from extra-pulmonary sites

**DOI:** 10.26508/lsa.202302076

**Published:** 2023-08-17

**Authors:** Tao Shi, Fenyong Shou, Ying He, Kan Zhou, Wenwan Gao, Xiaoping Nie, Mei Han, Chuanyu Liao, Tongxin Li

**Affiliations:** 1 Department of Orthopedics, Tianjin First Central Hospital , Tianjin, China; 2 Central Laboratory, Chongqing Public Health Medical Center, Chongqing, China; 3 Medical Department, Chongqing Public Health Medical Center, Chongqing, China

## Abstract

This study aimed to determinate characteristics of drug resistance *Mycobacterium tuberculosis* from patients with extra-pulmonary tuberculosis (EPTB). Patients were retrospectively studied from January 2020 to December 2021. All the isolates were cultured, tested drug susceptibility, and detected the gene mutation using whole genome sequencing. The correlations of whole genome sequencing, pattern of DR, patients’ distribution, and transmission were analyzed. 111 DR-EPTB isolates included pre–XDR-TB (53.2%), MDR-TB (29.7%), and poly–DR-TB (12.6%). The resistant drugs were INH followed by RFP and SM. The genotypes of 111 strains were lineage 2 and lineage 4. *KatG*_p.Ser315Thr was main gene mutation for resistance to INH; *rpsL*_p.Lys43Arg for SM, *rpoB*_p.Ser450Leu for rifampicin, *embB*_p.Met306Val for ethambutol, *gyrA*_p.Asp94Gly for FQs, and *pncA*_p.Thr76Pro for PZA. The residence was a significant risk factor for cluster transmission by patients and phenotypic DR types of strains for lineage 2 transmission. In the local area of southwest China INH, rifampicin and SM were main drugs in patients with DR-EPTB. *KatG*_p.Ser315, *rpoB*_p.Ser450Leu, and *rpsL*_p.Lys43Arg were main gene mutations. Phenotypic DR types and residence were main risk of transmission.

## Abstract (precis)

The article summarizes the characteristics of patients with drug resistance extra-pulmonary tuberculosis (DR-EPTB) and gene mutations of drug resistant *Mycobacterium tuberculosis* (DR-TB) from the patients with EPTB in a municipality of southwest China.

## Introduction

Tuberculosis is a threat to human health caused by *Mycobacterium tuberculosis* that affected lung and other extra-pulmonary sites. There was an estimated 10.0 million people fell ill with TB, 1.2 million TB deaths among HIV-negative people, and an additional 251,000 deaths among HIV-positive people in 2018 (World Health Organization, 2017). About a quarter of the world’s population is infected with M. tuberculosis (World Health Organization, 2020). Globally, an estimated 10.0 million (range, 8.9–11.0 million) people fell ill with TB in 2019 (World Health Organization, 2020). China is the third high burden country and account for 8.4% of global total (World Health Organization, 2020).

Extra-pulmonary tuberculosis (EPTB) is a common disease of TB, and sites of disease include lymph node, genitourinary, central nerve system, gastrointestinal, pleura, bone and joints, skin, and pericardial. According to the WHO Global Tuberculosis Report 2015, 32,000 cases of EPTB were diagnosed in nearly 530,000 new cases in China in 2014 (World Health Organization, 2015). EPTB is a growing public health concern in China, but data on drug resistance are limited, especially in Chongqing of southwest China. Although there were some research studies about epidemiology of spinal tuberculosis (Shi et al, 2016), molecular characteristic of MTB (Zhang et al, 2013), and drug resistance MTB (Shi et al, 2018) in Chongqing, there was little report about characteristics of DR-EPTB. Chongqing is a municipality city in southwest China, and the climate is wet and lack sunlight, which is suitable for reproduction and growth of MTB.

During the COVID-19 pandemic, some information was not reported in time. According to the WHO reports, an estimated 10.6 million people fell ill with TB worldwide in 2021, an increase of 4.5% from 10.1 million in 2020, and the TB incidence rate (new cases per 100,000 population per year) is estimated to have increased by 3.6% between 2020 and 2021, following declines of about 2% per year for most of the past two decades (World Health Organization, 2022). There was also no detail about TB incidence and new cases in China during the COVID-19 pandemic. This study determines the mutation characteristics and transmission of DR-EPTB from patients with EPTB using whole genome sequencing (WGS) to analyze drug resistance *M. tuberculosis* from patients with EPTB.

## Results

### Study population

**Figure 1.**
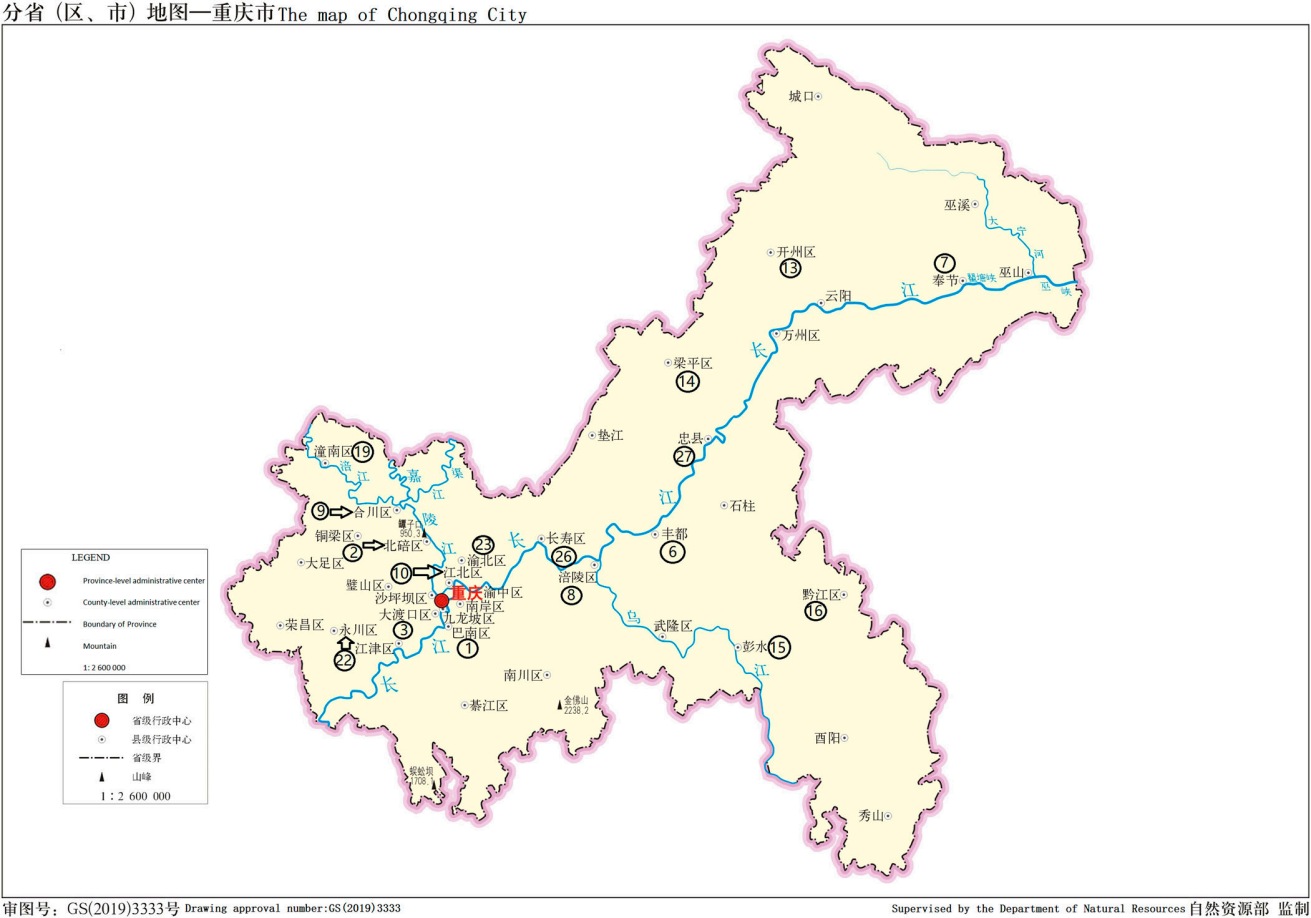
Regions of ≥3 patients in Chongqing city, the names of region represented by the circled number are in Table 2, the arrows link the circled number and names of region. The map is from the website: http://bzdt.ch.mnr.gov.cn/.

**Figure 2.**
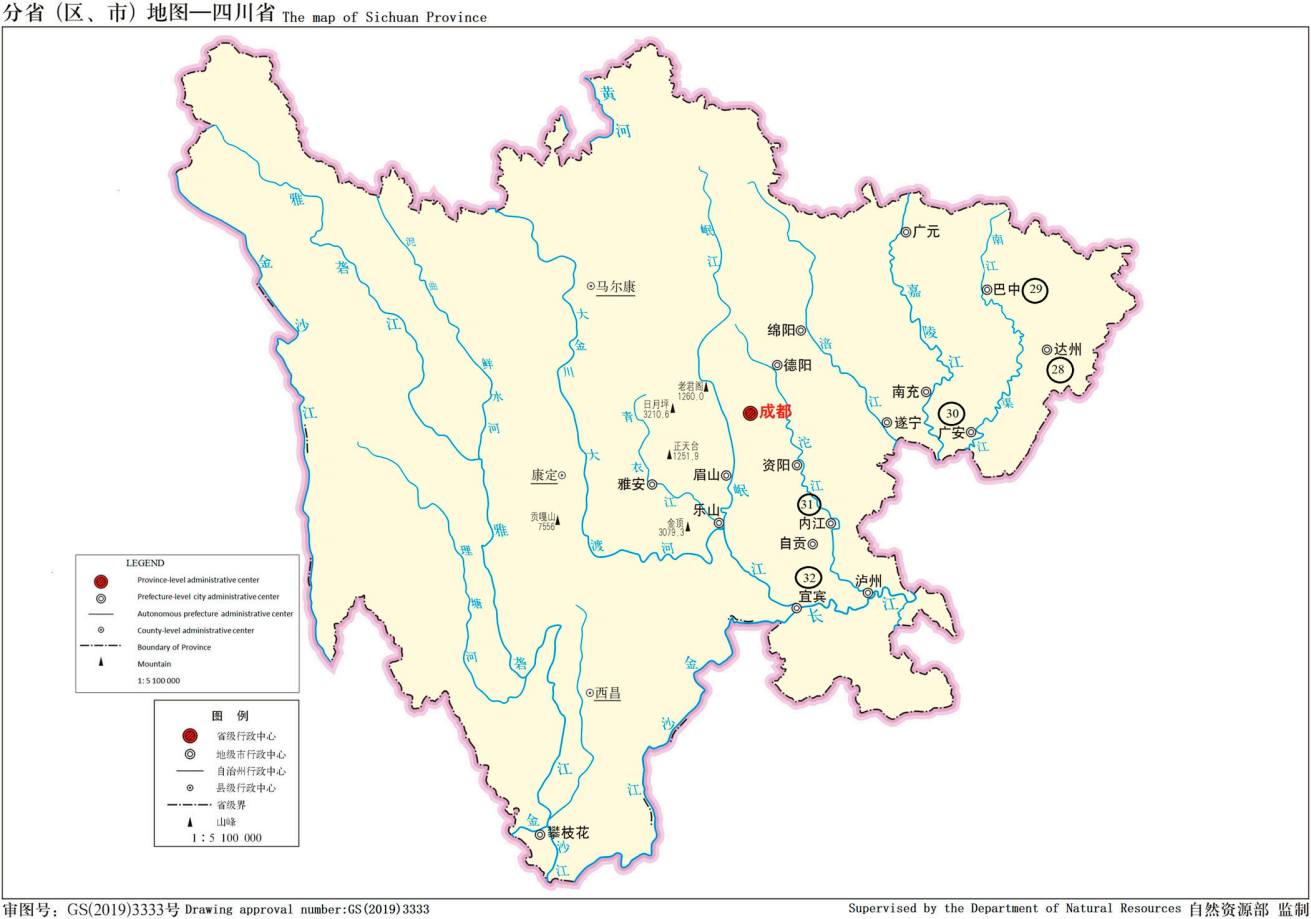
Regions of >1 patient in Sichuang Province, the names of the region represented by the circled number are in Table 2. The map is from the website: http://bzdt.ch.mnr.gov.cn/.

**Figure 3.**
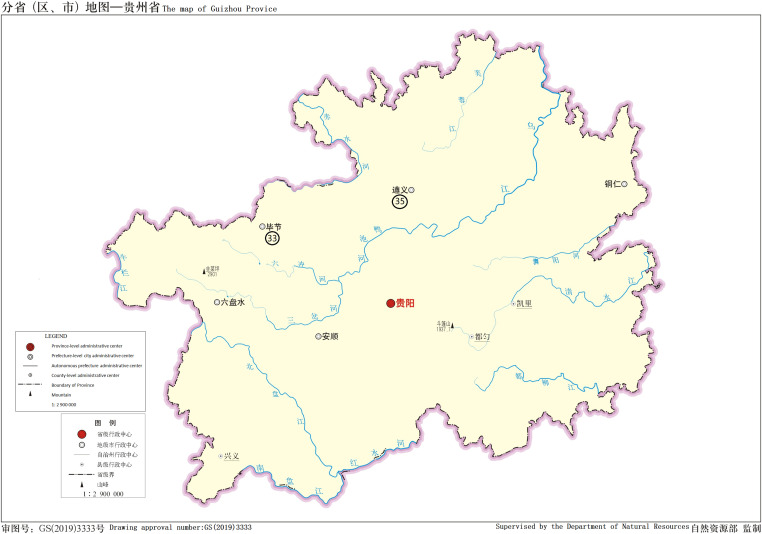
Regions of >1 patient in Guizhou Province, the names of the region represented by the circled number are in Table 2. The map is from the website: http://bzdt.ch.mnr.gov.cn/.

**Figure 4.**
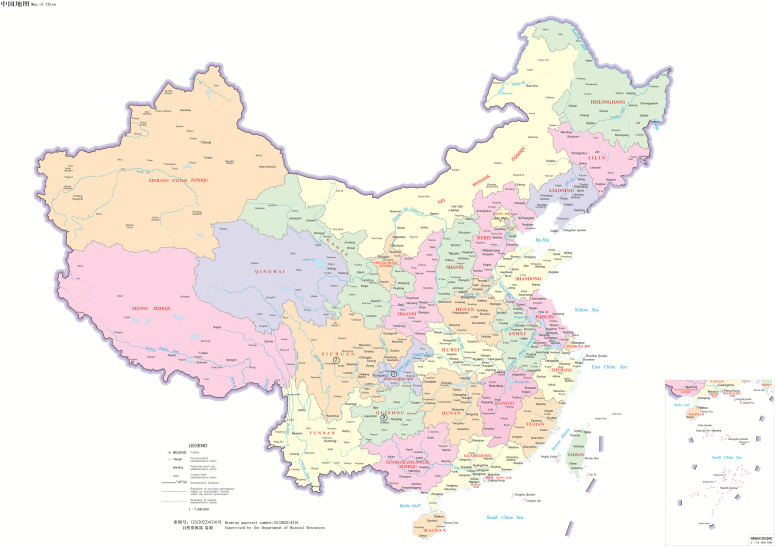
The three provinces are neighborhood regions. “1” with a circle stands for Chongqing city, “2” with a circle stands for Sichuan Province, “3” with a circle stands for Guizhou Province. The map is from the website: http://bzdt.ch.mnr.gov.cn/.

There were 111 patients including 71 male and 40 female, accounting for 64.0% and 36.0%, respectively. The age ranged from 13 to 75 yr. In the samples, the cold abscess accounted for 35.1%, pleural fluid 32.4%, and cerebrospinal fluid 11.7%. The new cases accounted for 60.4% and retreatment cases 39.6%. Table 1 displays the details. 9 of 111 patients had AIDS. Table 2 displays the number of patients from different regions. The major distribution of patients at the Chongqing city, Sichuan, and Guizhou Province is shown in the Figs 1–3. The locations of the three regions displayed in Fig 4 are Chongqing city, Sichuan Province, and Guizhou Province. (The geographical maps were downloaded at http://bzdt.ch.mnr.gov.cn/). There were no significant differences on the gender distribution in the three regions and patients’ types (Table 1).

**Table 1.** Demographic characteristic of 111 patients with DR-EPTB.

Characteristics	Value	*P*
Mean age (yr)	42.92 ± 17.99	
Sex, M/F	70/41	
Samples		
Lymph node (M/F)	2 (2/0)	
Pleural fluid (M/F)	36 (28/8)	
Urine (M/F)	7 (5/2)	
Cold abscess (M/F)	39 (17/22)	
Peritoneal fluid (M/F)	2 (1/1)	
Cerebrospinal fluid (M/F)	13 (9/4)	
Puncture fluid (M/F)	1 (1/0)	
Stool (M/F)	8 (6/2)	
Secreta (M/F)	2 (0/2)	
Others (M/F)	1 (1/0)	
Patients		
New cases (M/F)	67 (39/28)	>0.05
Retreatment (M/F)	44 (31/13)	
Regions		
Chongqing city (M/F)	87 (57/30)	>0.05
Sichuan Province (M/F)	16 (9/7)	
Guizhou Province (M/F)	8 (4/4)	

**Table 2.** Number of patients from different regions (n = 111).

Chongqing City (n = 87, 78.4%)		Sichuan Province (n = 16, 14.4%)		Guizhou Province (n = 8, 7.2%)	
regions	no. of patient	regions	no. of patient	regions	no. of patient
Banan District	2	Dazhou City	8	Bijie City	4
Beibei District	3	Bazhong City	1	Tongren City	1
Dadukou District	4	Guangan City	5	Zunyi City	3
Dazu District	1	Neijiang City	1		
Dianjiang County	2	Yibin City	1		
Fengdu County	5				
Fengjie County	3				
Fuling District	3				
Hechuan District	2				
Jiangbei District	6				
Jiangjin District	1				
Jiulongpo District	1				
Kaizhou District	2				
Liangping District	2				
Pengshui County	18				
Qianjiang District	3				
Shapingba Disdtrict	1				
Tongliang District	1				
Tongnan District	2				
Wulong District	1				
Xiushan County	1				
Yongchuan District	3				
Yubei District	2				
Yuzhong District	1				
Yunyang County	1				
Changshou District	6				
Zhong County	10				

### Phenotypic and genotypic drug susceptibility testing (DST)

**Figure 5.**
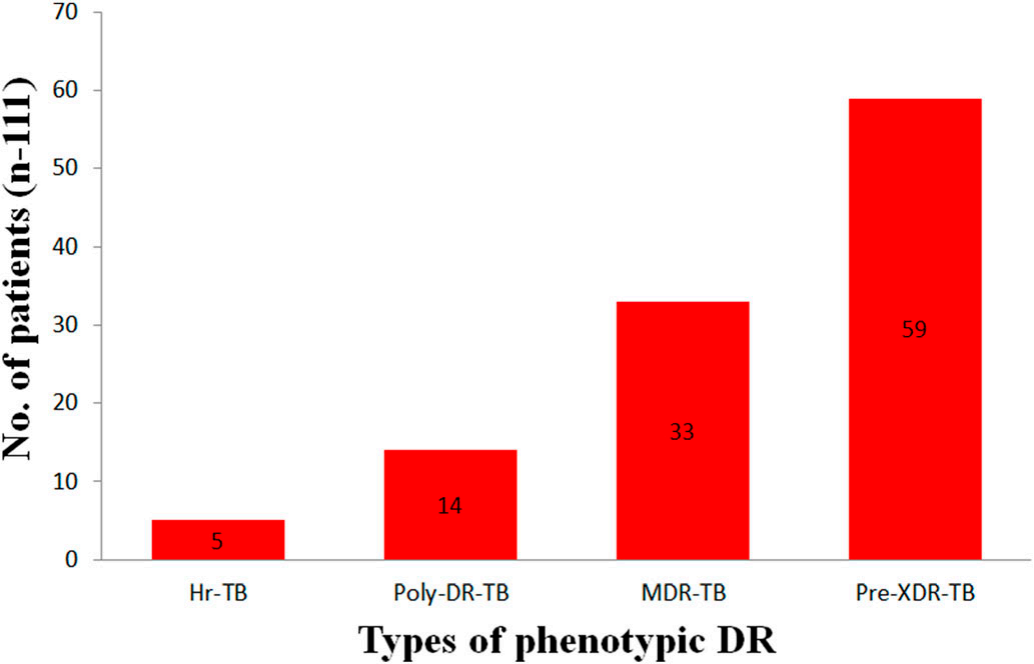
Number of patients with different types of DR.

**Figure 6.**
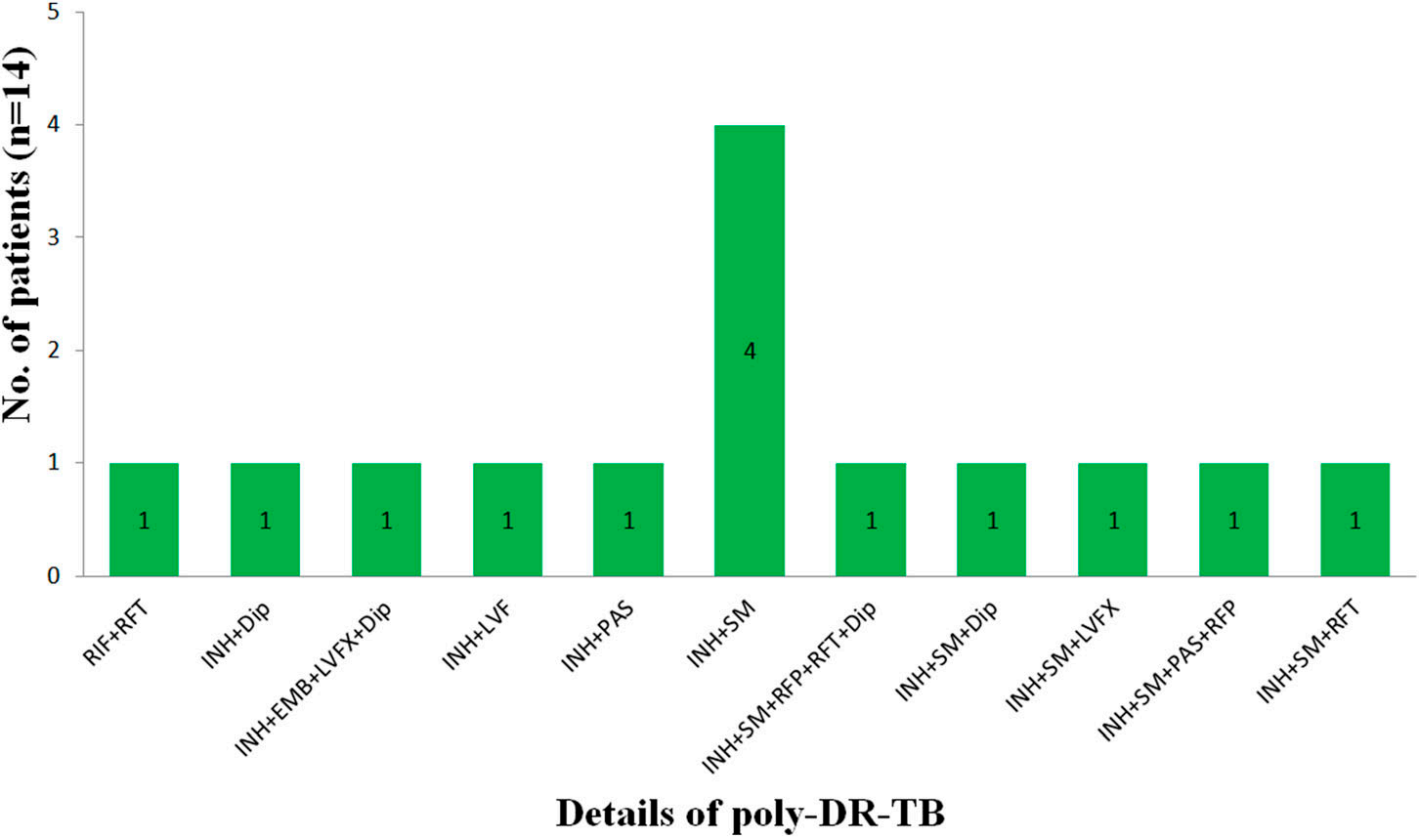
Number of patients with poly–DR-TB.

The pre–XDR-TB samples accounted for 53.2%, MDR-TB 29.7%, and poly–DR-TB 12.6% (Fig 5). There were five patients with HR-TB. The number of patients with poly–DR-TB except for MDR-TB, pre–XDR-TB, and HR-TB are shown in Fig 6. The drug with the highest number of resistance was INH followed by RFP and rifapentine (RFT), the fewest was capreomycin.

**Figure 7.**
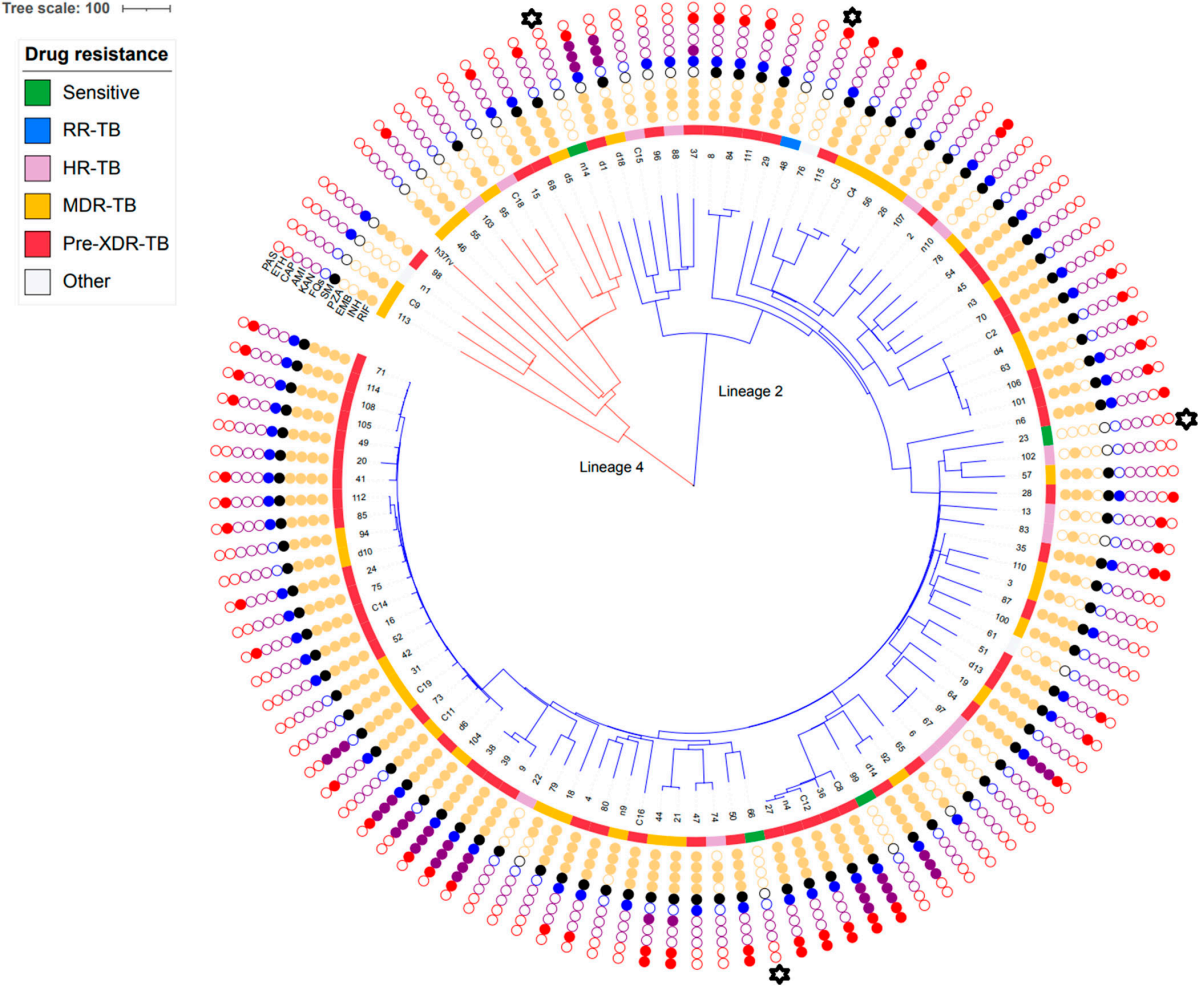
Dendrogram of 111 strains. 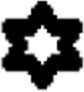
 display four strains sensitive strains in the results of whole genome sequencing for genotypic drug susceptibility, but phenotypic DR.

There were four sensitive strains in the results of WGS for genotypic drug susceptibility. The phenotypic DST of four strains was two MDR-TB, one HR-TB, and one poly–DR-TB. The numbers of the four genotypic sensitive strains were n14, 76, 23, and 66 (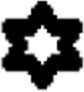 in the Fig 7 displays these four strains).

### WGS for phyletic evolution

A dendrogram of 111 DR-TB strains lineages is shown in Fig 7. A total of 98 isolates belonged to lineage 2 (East Asian), and the rest belonged to lineage 4 (Euro-American). There were 32 clusters (ranging from 2 to 4 isolates) including 61 isolates and 50 unique patterns (Fig 7). The cluster rate was 55.0%, and clustering ratio was 39.0%.

### WGS for gene variant

Of 111 strains, 90 contained gene mutations that were resistance to rifampicin (RIF), 101 to INH, 61 to PZA, 79 to ethambutol (EMB), 87 to SM, 59 to FQs, 15 to aminoglycosides, 44 to ethionamide (ETO), and 16 to para-aminosalicylic acid (PAS).

There were many gene mutations in the DR-TB in this study. *RpoB*_p.Ser450Leu accounted for 66.7% in the gene mutations resistance to RIF; *katG*_p.Ser315Thr for 93.1% to INH; *pncA*_p.Thr76Pro for 37.7% to PZA; *embB*_p.Met306Val and *embB*_p.Met306Ile for 43.0% and 27.8% to EMB, respectively; *rpsL*_p.Lys43Arg for 85.1% to SM; *gyrA*_p.Asp94Gly and *gyrA*_p.Asp94Ala for 40.7% and 20.3% to FQs, respectively; *rrs*_n.1401A>G for 73.3% to aminoglycosides; *fabG1*_c.-15C>T and *fabG1*_c.-8T>C for 27.3% and 25.0% to ETO, respectively; *folC*_p.Ile43Thr and *thyA*_p.His75Asn for 31.3% and 25.0% to PAS, respectively. Table 3 displays the details. Gene mutation appearing in only one strain is displayed in Table 4.

**Table 3.** The mutant genes of number of DR strains ≥2 (n = 111).

Drugs	Gene variant	No. of strains
RIF	*rpoB*_p.Ser450Leu	60
	*rpoB*_p.Asp435Gly	9
	*rpoB*_p.Leu430Pro	5
	*rpoB*_p.His445Asp	4
	*rpoC*_p.Gly332Arg	3
	*rpoB*_p.His445Cys	2
	*rpoB*_p.His445Gln	2
	*rpoB*_p.Thr427Ala	2
	*rpoC*_p.Leu527Val	2
	*rpoB*_p.Ala286Val	2
INH	*katG*_p.Ser315Thr	94
	*fabG1*_c.-8T>C	11
	*fabG1*_c.-15C>T	11
	*katG*_p.Ile317Val	2
	*katG*_p.Ser315Asn	2
PZA	*pncA*_p.Thr76Pro	23
	*pncA*_c.407dupA	6
	*pncA*_p.Gln141Pro	5
	*pncA*_c.390_391dupGG	2
	*pncA*_p.Pro62Leu	2
	*pncA*_p.Gln10Pro	2
EMB	*embB*_p.Met306Val	34
	*embB*_p.Met306Ile	22
	*embB*_p.Gln497Arg	7
	*embA*_c.-16C>G	6
	*embB*_p.Met306Leu	4
	*embB*_p.Asp354Ala	3
	*embA*_c.-16C>T	3
	*embB*_p.Gly406Ala	3
	*embB*_p.Gln497Pro	2
	*embB*_p.Gly406Asp	2
SM	*rpsL*_p.Lys43Arg	74
	*rpsL*_p.Lys88Arg	4
	*rrs*_n.514A>C	4
	*gid*_c.102delG	2
FQs	*gyrA*_p.Asp94Gly	24
	*gyrA*_p.Asp94Ala	12
	*gyrA*_p.Ala90Val	9
	*gyrA*_p.Asp94Asn	7
	*gyrA*_p.Asp94Tyr	4
	*gyrB*_p.Asp461Asn	2
Aminoglycosides	*rrs*_n.1401A>G	11
	*eis*_c.-10G>A	3
ETO	*fabG1*_c.-15C>T	12
	*fabG1*_c.-8T>C	11
	*ethA*_c.299delT	5
	*ethA*_c.1465dupG	5
	*ethA*_c.805dupC	4
	*ethA*_p.Arg421*	3
	*ethA*_c.9delG	2
	*ethA*_c.229_245dupGACGGCAAGCCCATCCT	2
PAS	*folC*_p.Ile43Thr	5
	*thyA*_p.His75Asn	4
	*folC*_p.Arg49Trp	2

**Table 4.** Gene mutation appearing in only one strain.

Drugs	Gene variant
RIF	*rpoB*_c.1297_1299dupTTC, *rpoB*_p.Gln429Leu, *rpoB*_p.His445Arg
	*rpoB*_p.His445Ser, *rpoB*_p.His445Tyr, *rpoB*_p.Ile491Phe
	*rpoB*_p.Phe424Leu, *rpoB*_p.Ser441Gln
INH	*fabG1*_c.-17G>T, *inhA*_c.-154G>A, *katG*_c.1002dupG
PZA	*pncA*_c.-1110_448del, *pncA*_c.-11A>G, *pncA*_c.-12T>C
	*pncA*_c.287dupA, *pncA*_p.Thr76Pro, *pncA*_c.417dupG, *pncA*_p.Cys14*
	*pncA*_c.491dupC, *pncA*_p.Ala171Thr, *pncA*_p.Asp12Ala, *pncA*_p.Ile6Thr
	*pncA*_p.Cys14Arg, *pncA*_p.Gly132Asp, *pncA*_p.Gly17Asp, *pncA*_p.Val139Ala
	*pncA*_p.His82Arg, *pncA*_p.Leu159Arg, *pncA*_p.Leu172Pro, *pncA*_p.Val139Leu
	*pncA*_p.Ser67Pro, *pncA*_p.Trp119Arg, *pncA*_p.Tyr103Cys
EMB	*embA*_c.-11C>A, *embB*_p.Tyr319Cys, *embB*_p.Arg274Pro
	*embB*_p.Asp1024Asn, *embB*_p.Asp328Gly
SM	*gid*_c.115delC, *gid*_p.Glu103*, *rpsL*_p.Lys88Met, *rrs*_n.517C>T
FQs	*gyrB*_p.Glu501Asp, *gyrA*_p.Asp94His
Aminoglycosides	*rrs*_n.1402C>A
ETO	*ethA*_c.11dupA, *ethA*_c.341dupA, *ethA*_c.364dupA, *ethA*_c.567dupG
	*ethA*_c.578dupC, *ethA*_c.672dupG, *ethA*_c.752dupG, *ethA*_c.924dupG
	*ethA*_p.Tyr461*, *ethA*_c.1431delT, *inhA*_c.-154G>A
PAS	*folC*_p.Glu153Ala, *folC*_p.Ser150Gly, *thyA*_p.Arg235Pro, *thyX*_c.-16C>T
	*thyA*_p.Trp101Arg

### Relationships between gene mutation and patients’ living regions

There were seven regions where patients’ number was more than 5. Pengshui was the region of the most patients, followed by Zhong County and Dazhou City. The patients’ number with different gene mutations in different regions is displayed in Table 5. In the seven regions, there were 58 patients with DR-EPTB that accounted for 52.3% of 111 patients. Of 58 patients, *katG*_p.Ser315Thr accounted for 87.49% of resistance to INH; *rpsL*_p.Lys43Arg for 74.1% to SM; *rpoB*_p.Ser450Leu for 62.1% to RIF; *embB*_p.Met306Val, *embB*_p.Met306Ile, and *embB*_p.Met306Leu for 37.9%, 12.1%, and 8.6% to EMB, respectively; *gyrA*_p.Asp94Gly, *gyrA*_p.Asp94GlyAla, *gyrA*_p.Asp90Val, and *gyrA*_p.Asp94Asn for 20.7%, 15.5%, 6.9%, and 5.2% to FQs, respectively; *pncA*_p.Thr76Pro for 31.0% to PZA.

**Table 5.** Gene variant analysis of regions of number of patients with DR strains ≥5.

Regions	No. of patients	Drugs	Gene variant	No. of strains
Pengshui County	18	RIF	*rpoB*_p.Ser450Leu	16
			*rpoC*_p.Leu527Val	2
		INH	*katG*_p.Ser315Thr	17
			*fabG1*_c.-8T>C	1
		PZA	*pncA*_p.Thr76Pro	15
			*pncA*_p.Ser67Pro	1
			*pncA*_c.390_391dupGG	1
		EMB	*embB*_p.Met306Val	16
			*embB*_p.Gln497Arg	1
			*embB*_p.Arg274Pro	1
		SM	*rpsL*_p.Lys43Arg	16
		FQs	*gyrA*_p.Asp94Ala	6
			*gyrA*_p.Asp94Gly	3
			*gyrA*_p.Asp94Asn	1
		Aminoglycosides	*rrs*_n.1401A>G	2
		ETO	*ethA*_c.805dupC	4
			*ethA*_c.1465dupG	1
			*ethA*_c.924dupG	1
			*ethA*_c.578dupC	1
			*fabG1*_c.-8T>C	1
Zhong County	10	RIF	*rpoB*_p.Ser450Leu	4
			*rpoB*_p.Asp435Val	2
			*rpoB*_p.Thr427Ala	2
			*rpoB*_p.Asp435Tyr	1
			*rpoB*_p.Leu430Pro	1
			*rpoB*_c.1297_1299dupTTC	1
		INH	*katG*_p.Ser315Thr	9
			*fabG1*_c.-15C>T	2
		PZA	*pncA*_p.Thr76Pro	1
			*pncA*_c.491dupC	1
			*pncA*_p.Leu172Pro	1
			*pncA*_c.407dupA	1
			*pncA*_p.Cys14*	1
		EMB	*embB*_p.Met306Val	3
			*embA*_c.-16C>G	2
			*embB*_p.Met306Ile	1
			*embB*_p.Asp354Ala	1
			*embA*_c.-11C>A	1
		SM	*rpsL*_p.Lys43Arg	8
			*rpsL*_p.Lys88Arg	1
		FQs	*gyrA*_p.Asp94Ala	2
			*gyrA*_p.Asp94Gly	2
			*gyrA*_p.Ala90Val	1
		ETO	*fabG1*_c.-15C>T	2
			*ethA*_c.299delT	1
		PAS	*thyA*_p.His75Asn	2
			*folC*_p.Ile43Thr	1
Dazhou City	8	RIF	*rpoB*_p.Ser450Leu	4
			*rpoB*_p.Asp435Val	1
			*rpoB*_p.Gln429Leu	1
			*rpoC*_p.Gly332Arg	1
			*rpoB*_p.Leu430Pro	1
			*rpoB*_p.Asp435Gly	1
		INH	*katG*_p.Ser315Thr	8
			*fabG1*_c.-15C>T	1
			*fabG1*_c.-8T>C	1
		PZA	*pncA*_p.Thr76Pro	2
			*pncA*_p.Gln10Pro	1
			*pncA*_c.-11A>G	1
			*pncA*_c.-12T>C	1
		EMB	*embB*_p.Met306Val	3
			*embB*_p.Met306Ile	2
			*embB*_p.Met306Leu	1
		SM	*rpsL*_p.Lys43Arg	6
		FQs	*gyrA*_p.Asp94Gly	3
			*gyrA*_p.Ala90Val	1
		ETO	ethA_c.9delG	2
			*ethA*_c.1465dupG	1
			*fabG1*_c.-15C>T	1
			*fabG1*_c.-8T>C	1
Changshou District	6	RIF	*rpoB*_p.Ser450Leu	4
			*rpoB*_p.Asp435Gly	1
		INH	*katG*_p.Ser315Thr	5
			*fabG1*_c.-8T>C	1
		PZA	*pncA*_p.Gln141Pro	2
			*pncA*_p.Leu159Arg	1
		EMB	*embB*_p.Met306Leu	2
			*embB*_p.Gly406Asp	1
			*embB*_p.Gln497Arg	1
		SM	*rpsL*_p.Lys43Arg	3
		FQs	*gyrA*_p.Asp94Gly	2
			*gyrA*_p.Ala90Val	1
			*gyrA*_p.Asp94Ala	1
		Aminoglycosides	*rrs*_n.1401A>G	2
		ETO	*ethA*_c.1465dupG	2
			*fabG1*_c.-8T>C	1
Jiangbei District	6	RIF	*rpoB*_p.Ser450Leu	5
		INH	*katG*_p.Ser315Thr	5
			*fabG1*_c.-15C>T	1
		PZA	*pncA*_p.Pro62Leu	2
			*pncA*_c.390_391dupGG	1
			*pncA*_p.Thr76Pro	1
		EMB	*embB*_p.Met306Ile	2
			*embB*_p.Met306Val	2
			*embA*_c.-16C>T	1
		SM	*rpsL*_p.Lys43Arg	4
			*rpsL*_p.Lys88Arg	1
			*gid*_c.102delG	1
		FQs	*gyrA*_p.Asp94Gly	2
			*gyrA*_p.Asp94Asn	1
		Aminoglycosides	*rrs*_n.1401A>G	1
		ETO	*fabG1*_c.-15C>T	1
		PAS	*folC*_p.Ser150Gly	1
Fengdu County	5	RIF	*rpoB*_p.Ser450Leu	2
			*rpoB*_p.Asp435Val	1
			*rpoB*_p.His445Asp	1
			*rpoB*_p.His445Arg	1
		INH	*katG*_p.Ser315Thr	4
			*fabG1*_c.-15C>T	1
		PZA	*pncA*_c.407dupA	1
			*pncA*_p.Tyr103Cys	1
		EMB	*embB*_p.Met306Ile	2
			*embB*_p.Gln497Pro	1
			*embA*_c.-16C>G	1
		SM	*rpsL*_p.Lys43Arg	3
		FQs	*gyrA*_p.Ala90Val	2
			*gyrA*_p.Asp94Asn	1
		Aminoglycosides	*rrs*_n.1401A>G	1
		ETO	*ethA*_c.299delT	1
			*fabG1*_c.-15C>T	1
		PAS	*folC*_p.Ile43Thr	1
Guangan City	5	RIF	*rpoB*_p.Ser450Leu	1
			*rpoB*_p.Asp435Gly	1
			*rpoB*_p.Leu452Pro	1
		INH	*katG*_p.Ser315Thr	3
			*fabG1*_c.-8T>C	1
		PZA	*pncA*_c.-1110_448del	1
		EMB	*embB*_p.Gln497Arg	1
		SM	*rpsL*_p.Lys43Arg	3
		ETO	*fabG1*_c.-8T>C	1

### Transmission of EPTB in the local area

The risk factors for recent transmission were analyzed. The results of univariate analysis showed that residence was significantly associated with disease transmission (*P* ≤0.01), and the phenotypic DR types of strains was in significant correlation with lineage 2 (*P* ≤0.05) (Table 6). The gender, age, residence, types of patients, DR types of strains, and patients’ living regions were progressed to multivariate analysis (Table 7). MDR-TB and poly–DR-TB were the risk factors of infecting lineage 2 strains. There was no correlation between the lineage and cluster (Table 8).

**Table 6.** Univariate analysis of the risk factor for recent transmission among patients (n = 111).

	Lineage (n = 111)				Cluster (n = 111)			
	lineage 2 (n = 98)	lineage 4 (n = 13)	χ^2^	*P*	non-cluster (n = 50)	cluster (n = 61)	χ^2^	*P*
Gender								
Male	59	11	2.936	>0.05	32	38	0.034	>0.05
Female	39	2			18	23		
Age (years)								
≤30	33	5	0.155	>0.05	19	19	0.835	>0.05
31–60	43	5			21	26		
≥61	23	3			10	16		
Residence								
Rural	80	8	2.534	>0.05	47	40	13.102	<0.01
Urban	19	5			3	21		
Samples								
Lymph node	2	0	11.188	>0.05	2	0	13.571	>0.05
Pleural fluid	32	4			10	26		
Urine	5	2			4	3		
Cold abscess	36	3			19	20		
Peritoneal fluid	2	0			2	0		
Cerebrospinal fluid	11	2			6	7		
Puncture fluid	1	0			0	1		
Stool	7	1			3	5		
Secreta	2	0			0	2		
Others	0	1			1	0		
Types of patient								
New	61	6	1.242	>0.05	28	39	0.723	>0.05
Retreatment	37	7			22	22		
Phenotypic DR types of strains								
HR-TB	4	1	9.468	<0.05	1	4	6.317	>0.05
MDR-TB	27	6			10	23		
Poly–DR-TB	10	4			7	7		
Pre–XDR-TB	57	2			32	27		
Patients’ living regions								
Chongqing city	78	9	0.929	>0.05	41	46	0.704	>0.05
Sichuan Province	13	3			6	10		
Guizhou Province	7	1			3	5		
Genotypic DR types of strains								
INH (n = 101)	91	10	13.892	>0.05	45	56	6.708	>0.05
SM (n = 87)	83	4			40	47		
RIF (n = 90)	81	9			39	51		
EMB (n = 79)	75	4			33	46		
PAS (n = 16)	16	0			11	5		
RFT (n = 101)	91	10			45	56		
1321Th (n = 12)	12	0			7	5		
CPM (n = 12)	9	3			5	7		
FQs (n = 59)	55	4			27	32		
Aminoglycosides (n = 15)	15	0			8	7		
Dip (n = 70)	65	5			29	41		
PZA (n = 61)	58	3			28	33		
ETO (n = 44)	41	3			23	21		

**Table 7.** Multivariable Logistic Regression for risk factor of recent transmission in patients.

	Lineage		Cluster	
	OR (95% CI)	*P*-value	OR (95% CI)	*P*-value
Gender				
Male	0.224 (0.039–1.271)	0.091	0.956 (0.412–2.215)	0.916
Female	Ref.		Ref.	
Age (years)				
≤ 30	Ref.		Ref.	
31–60	1.995 (0.378–10.537)	0.416	1.438 (0.586–3.530)	0.428
≥61	2.444 (0.348–17.153)	0.369	2.079 (0.672–6.438)	0.204
Residence				
Rural	Ref.		Ref.	
Urban	1.254 (0.242–6.494)	0.788	1.610 (0.617–4.197)	0.330
Types of patient				
New	4.551 (0.966–21.433)	0.055	1.66 (0.709–3.911)	0.242
Retreatment	Ref.		Ref.	
Phenotypic DR types of strains				
HR-TB	0.069 (0.004–1.279)	0.073	0.499 (0.072–3.482)	0.483
MDR-TB	0.096 (0.015–0.605)	0.013	0.903 (0.363–2.242)	0.825
Poly–DR-TB	0.061 (0.008–0.501)	0.009	0.626 (0.181–2.168)	0.460
Pre–XDR-TB	Ref.		Ref.	
Patients’ living regions				
Chongqing city	Ref.		Ref.	
Sichuan Province	0.522 (0.085–3.207)	0.483	1.582 (0.492–5.091)	0.442
Guizhou Province	2.163 (0.136–34.292)	0.584	2.097 (0.422–10.415)	0.365

**Table 8.** The correlation between the linage and cluster.

	Cluster (n = 61)	Non-cluster (n = 50)	χ^2^	*P*
Lineage 2 (n = 98)	53	45	0.2578	>0.05
Lineage 4 (n = 13)	8	5		

In conclusion, residence was a significant risk factor for cluster transmission by patients and phenotypic DR types of strains for lineage 2 transmission.

### Analysis of the highest incidence local area and a typical case

**Figure 8.**
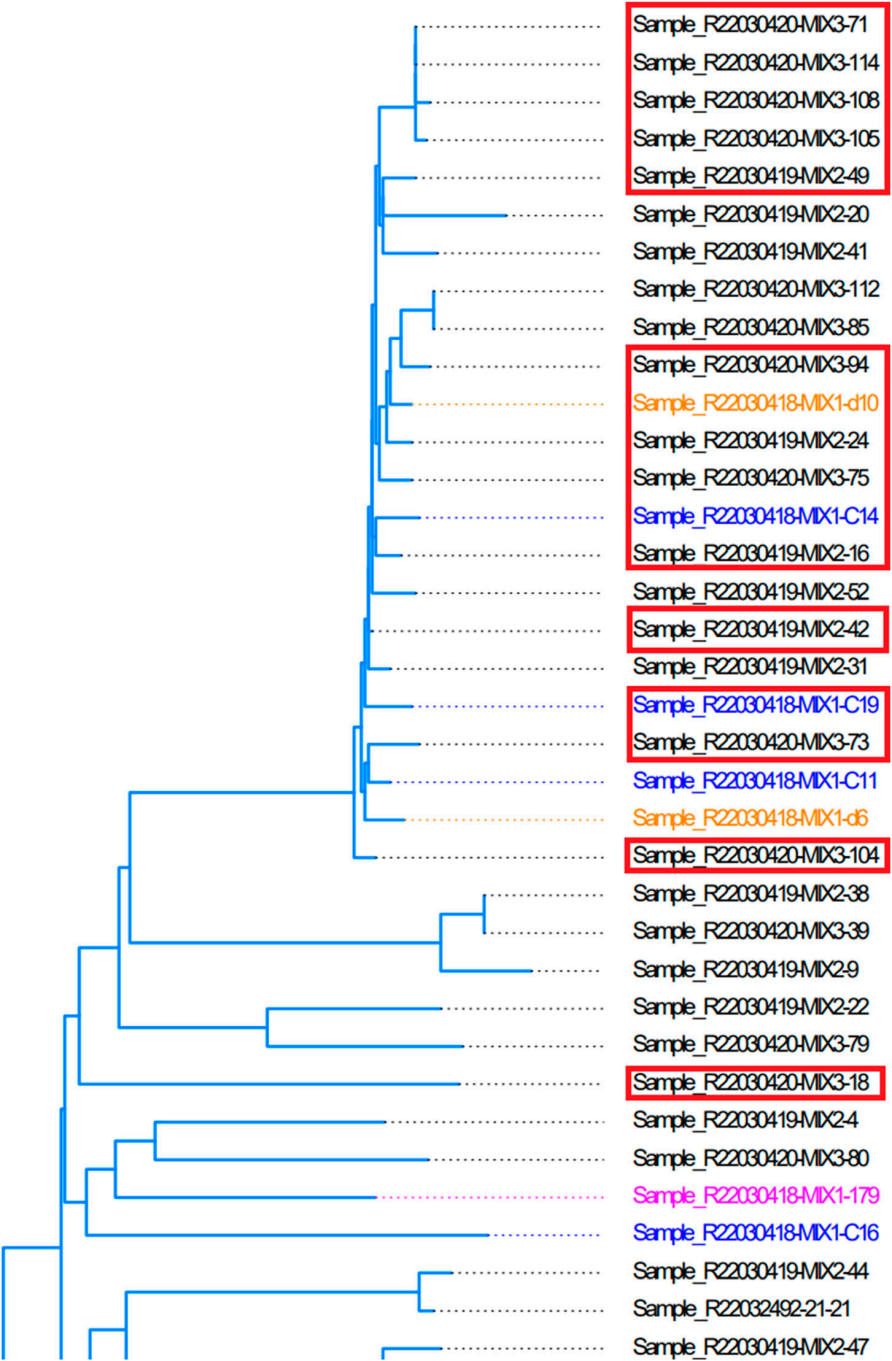
Phyletic evolution of strains from Pengshui County of Chongqing City.

Pengshui County was the highest incidence area in this study. There were 18 patients enrolled in the study. Of 18 patients, nine patients lived in the same street and town, and the samples were R22030420-MIX3-108, R22030420-MIX3-71, R22030420-MIX2-49, R22030420-MIX3-18, R22030420-MIX1-d10, R22030420-MIX1-C19, R22030420-MIX3-75, R22030420-MIX3-104, R22030420-MIX2-42. (The numbers of samples in Fig 7 were 108, 71, 49, 18, d10, C19, 75, 104, and 42). The other nine patients lived in the four neighboring towns. There were 16 strains that were close in the phylogenetic tree (Fig 8). 18 patients included 5 retreatment and 13 new cases. Five retreatment strains contained three MDR-TB and two pre-XDR-TB. 13 new strains contained 1 poly–DR-TB, 3 MDR-TB, and 9 pre-XDR-TB.

There was a typical patient who was hospitalized twice. The time interval was 4 mo. The first hospitalization time was 25 Apr 2020. The first sample from patient was pleural fluid. The phenotypic resistant drugs included INH + SM + RFP + EMB + RFT. The gene mutations were *rpoB*_p.Ser450Leu for resistance to RFP, *fabG1*_c.-15C>T to INH, *pncA*_p.Leu172Pro to PZA, *embB*_p.Met306Val to EMB, *rpsL*_p.Lys43Arg to SM, *gyrA*_p.Asp94Ala to FQs, and *fabG1*_c.-15C>T to ETO. The second hospitalization time was 26 Aug 2020. The second sample from patient was cold abscess. The phenotypic resistant drugs included INH + SM + RFP + EMB + RFT + Dip. The gene mutations were *rpoB*_p.Ser450Leu for resistance to RFP, *katG_*p.Ser315Thr to INH, *pncA*_p.Thr76Pro to PZA, *embB*_p.Met306Val to EMB, *rpsL*_p.Lys43Arg to SM, and *gyrA*_p.Asp94Ala to FQs. The number of second resistant drug was one more than number of the first, which was dip. The gene mutations changed to *katG_*p.Ser315Thr to INH and *pncA*_p.Thr76Pro to PZA from the first mutation of *fabG1*_c.-15C>T to INH and *pncA*_p.Leu172Pro to PZA.

## Discussion

TB is a major cause of ill health and one of the leading causes of death worldwide. Until the coronavirus (COVID-19) pandemic, TB was the leading cause of death from a single infectious agent, ranking above HIV/AIDS (World Health Organization, 2022). The World Health Organization (WHO) has published a global TB report every year since 1997. To reach the milestones and targets for reductions in TB incidence required an annual decline in the TB incidence rate of 4–5% per year by 2,020, accelerating to 10% per year by 2,025, and then to an average of 17% per year from 2,025 to 20,357, and not only these declines in TB and EPTB incidence but also reductions in the DR incidence. The 30 high TB burden countries accounted for 87% of all estimated incident cases worldwide, and China accounted for 7.4%, rank 3. According to report, in China, the incidence of TB was 55 per 100,000 and MDR/RR was 2.3 per 100,000 in which new case accounted for 3.4% and retreatment for 19% (World Health Organization, 2022). There were many researches on WGS for gene mutations about DR pulmonary tuberculosis (DR-PTB), but a few on WGS for gene mutations about DR-EPTB, especially in the high incidence area of DR-PTB. This study focused on DR-EPTB of the above area.

In this study, the samples included 10 types in which the proportion of cold abscess was the highest followed by pleural fluid and cerebrospinal fluid. There were some studies about WGS for sample of EPTB, for example, 71 samples from tuberculous spondylitis in Russia (Chernyaeva et al, 2018), five samples from cerebrospinal fluid and joint aspirate pus and cervical lymph node in India (Sharma et al, 2017; Advani et al, 2018). The previous study focused on single-type sample, but this study included all type samples from extra-pulmonary sites. The aim of including all type samples in this study was to determinate the transmission of DRTB in the patients and gene mutations in the different type of samples. The statistical univariate analysis showed that the sample types were of no relationships with the clustering and different lineages. This result showed that sample type from extra-pulmonary site was no risk factor for transmission in the patients. But there was a case report about the nosocomial transmission of extra-pulmonary MTB infection (Walker et al, 2017). This report reminded that suppurating wounds in tuberculosis constituted a hazard requiring risk assessment for transmission. Although, in our study all the samples were collected in the operation room that was sterilized strictly between the different patients entering the room except for urine, stool, and secreta samples.

In the patients, new patients with DR were more than retreatment. But according to the report, the incidence of MDR/RR in retreatment was higher than new case (World Health Organization, 2022). The incidence of the report was average number from the whole country, maybe there was different incidences in the different regions of country. In this study, the patients were from three regions including Chongqing City that was a municipality at the southwest China, Sichuan Province, and Guizhou Province that were adjacent to Chongqing City. Patient in Chongqing City was the most. In Chongqing City, patients’ number from Pengshui County was the highest followed by Zhong County, Changshou District, and Jiangbei District. In Sichuang Province, patients’ number from Dazhou City was the highest followed by Guangan City. In Guizhou Province, patients’ number from Bijie City was the highest followed by Zunyi City. Sichuan Province locates at the northwest of Chongqing municipality, and Guizhou Province locates at the South of Chongqing municipality. Zunyi City and Bijie City locate at the north of Guizhou Province which was close to Chongqing municipality, and Dazhou City and Guangan City locate at the east of Sichuan Province which was also close to Chongqing municipality. There were more patients in the transition area of the three provinces and cities because of the large and frequent personnel flow that may be the reason of new DR patients more than retreatment.

WGS has been widely used for detection of DR-TB (Lee and Behr, 2016). There were many studies of PTB and DR-PTB using WGS to determinate gene mutations, DR characteristics, and transmission (Bang, 2010; Juma et al, 2019; Rkia & Mounsef, 2020; Reta et al, 2021; Mujuni et al, 2022). Also, there were some researches in China (Yang et al, 2018; Jiang et al, 2020; Zhao et al, 2022). All the above researches were on the DR-PTB, even studies on DR-EPTB the samples were limited (Sharma et al, 2017; Advani et al, 2018; Chernyaeva et al, 2018). The types of gene mutations were different in different countries and regions (Bang, 2010; Sharma et al, 2017; Walker et al, 2017; Advani et al, 2018; Chernyaeva et al, 2018; Yang et al, 2018; Juma et al, 2019; Jiang et al, 2020; Rkia & Mounsef, 2020; Reta et al, 2021; Mujuni et al, 2022; Zhao et al, 2022). According to the first nationwide drug-resistant tuberculosis surveillance program in China, more than half of MDR strains were resistant to at least five anti-tuberculosis drugs in each administrative region, especially in northern (22%), eastern (20%), northwest (26%), and southwest (29%) China, with more than 20% of MDR strains resistance to at least eight drugs (Huang et al, 2019). In Shenzhen City of China, *katG*315, *RpoB*450, *rpsL*43, *gyrA*94, and *rrs*514 were the main gene mutations (Jiang et al, 2020). In another research on Chongqing City of China, *embB*_p.Met306Ile, *embB*_p.Gly406Asp, *embB*_p.Gln497Arg, *embB*_p.Asp354Ala, *embB*_p.Met306Val, *rrs*_r.1401a>g, *fabG1*_c.-8T>C, and *fabG1*_c.-15C>T were the main gene mutations (Zhao et al, 2022). All the above researches were on the basis of sputum sample, but the research on EPTB was different. *katG*_p.S315T, *RpoB*_p.S450L, *rpsL*_p.K43R, *emb*A-16, *pncA*Y103Stop, *eis*-37, and *eis*-10 were the main gene mutations of EPTB samples from tuberculous spondylitis (Chernyaeva et al, 2018), whereas in India, five EPTB samples including CSF, joint aspirate pus, and cervical lymph node showed the main gene mutations including *embC* R738Q, *embB* G406S, *gyrA* E21Q, *gyrA* G668D, *gyrA* S95T, *fbpC* G158S, *katG* R463L, *rpoB* D435Y, *rpoB* L430P, *rpoB* L452P, and *gid* Q125*. From the above researches, it was concluded that the gene mutations were different from different regions and samples. In this study, more samples from extra-pulmonary sites were collected and detected using WGS. *RpoB*_p.Ser450Leu had the most gene mutation for RIF resistance, *katG*_p.Ser315Thr for INH, *pncA*_p.Thr76Pro for PZA, *gyrA*_p.D94G, *embB*_p.Met306Val and *embB*_p.Met306Ile for EMB, *rpsL*_p.Lys43Arg for SM, *gyrA*_p.Asp94Gly and *gyrA*_p.Asp94Ala for FQs, *rrs*_n.1401A>G for aminoglycosides, *fabG1*_c.-15C>T and *fabG1*_c.-8T>C for ETO, and *folC*_p.Ile43Thr and *thyA*_p.His75Asn for PAS. At the same time, the statistical univariate analysis showed that the genotypic DR types of strains had no relationships with the clustering and different lineages.

In this study of patients with EPTB, new cases were more than retreatment, which showed new patients infected DRTB at the first onset that invaded extra-pulmonary sites. The DRTB underwent transmission very seriously. In this study, the phenotypic DR types of strains were in relationship with lineage, and the residence was in correlation with clustering. Besides, the result of multivariable logistic regression displayed the phenotypic DR types of strains which was the risk factor of recent transmission in patients. This result showed the DRTB was in transmission in the rural seriously in the local area of southwest China. This study display that the types and frequency of gene mutations in the high incidence area was obviously higher than low incidence area.

In this study, there was a typical patient who was hospitalized twice. The number of resistant drugs was plus one in the second hospitalization than the first. The gene mutation was different between the two hospitalizations. The gene mutations changed to *katG_*p.Ser315Thr to INH and *pncA*_p.Thr76Pro to PZA from the first mutation of *fabG1*_c.-15C>T to INH and *pncA*_p.Leu172Pro to PZA. The *katG_*p.Ser315Thr and *pncA*_p.Thr76Pro were common gent mutations, but *fabG1*_c.-15C>T and *pncA*_p.Leu172Pro were seldom. There were a few reports about the research to determinate the reason. In our opinions, the reason was maybe that the drug resistance *M. tuberculosis* strains were different.

There were four strains that were different between phenotypic and genotypic DST. Four strains included 1 HR-TB, two MDR-TB, and one poly–DR-TB (INH + PAS). There were some researches about gene mutations including *RpoB*_p.Leu511Pro, *RpoB*_p.Leu533Pro, *RpoB*_p.Asp516Tyr, *RpoB*_p.His526Asn, *RpoB*_p.L430P, *RpoB*_p.D435Y, *RpoB*_p.L452P, and *RpoB*_p.H445C/L that lead the discordant result between phenotypic and genotypic DST (Rigouts et al, 2013; Paolo et al, 2018; Mvelase et al, 2019; Joseph et al, 2021). The reason of discordance was that phenotypic RIF resistance testing of *M. tuberculosis* is not a binary phenomenon for some *rpoB* mutations and that the widely used automated MGIT 960 system was prone to miss some RIF resistance–conferring mutations, whereas careful DST on LJ missed hardly any (Rigouts et al, 2013). Also the reason between phenotypic and genotypic DST for INH was maybe that the *katG* deletion was detected in 9.4% isolates studied, confirming previous reports from different geographic regions that this event in causing INH resistance was infrequent (Rouse et al, 1996). Besides, the report showed that 14 of 106 (13.2%) INH-resistant strains did not find any mutations in the genes of *katG315* and *inhA* promoter region, which indicated that there are other mechanisms of resistance or gene mutations external to these nucleotides in the clinical strains (Wu et al, 2006). The previous article had displayed the mechanisms of resistance to INH and ETH in detail (Vilchèze and Jacobs, 2014), but there maybe some other mechanisms that have not been found and need to be studied further.

In the local area of southwest China, INH, RIF, and SM were three main drugs in patients with DR-EPTB. KatG_p.Ser315, rpoB_p.Ser450Leu, and rpsL_p.Lys43Arg were main gene mutations. Phenotypic DR types and residence were the main risk of transmission. These analyses provide reference for the prevention and treatment of DR-EPTB in local area of southwest China.

## Materials and Methods

### Study population

111 cases of inpatients with DR-EPTB, who were hospitalized in Chongqing Public Health Medical Center from January 2020 to December 2021, were included in this study.

### Specimen collection and pretreatment

Specimens (pus and excised tissues) were collected during surgical procedures and stored at low temperature (4–8°C). Then, the specimens were decontaminated using the N-acetyl-L-cysteine–NaOH method. The processed sediment was washed using a sterile 0.9% NaCl solution, re-suspended in 1.5 ml sterile 0.9% NaCl solution, and then equally divided into three thirds.

### Specimen separation and culture

One of the three thirds was then centrifuged, and the sediment was inoculated in both BACTEC MGIT 960 system (Bacton Dickinson and Company) and neutral Roche medium. The culture was regarded as positive if one or both of the above two culture methods produced positive results.

### Phenotypic DST

Drug susceptibility testing was performed using the proportion method in the Roche medium recommended by the World Health Organization, and the concentrations of drugs in media were as follows: isoniazid (INH) 0.2 μg/ml, RIF 40 μg/ml, EMB 2 μg/ml, streptomycin (SM) 4 μg/ml, RFT 40 μg/ml, PAS 1.0 μg/ml, amikacin 30 μg/ml, capreomycin 40 μg/ml, levofloxacin (LVFX) 2 μg/ml, protionamide (1321Th) 40 μg/ml, and dipasic (Dip) 0.1 μg/ml. A strain was declared resistant to a drug when the growth rate was >1% compared with the control. HR-TB was defined as mono-isoniazid resistant, RIF-susceptible TB. RR-TB was defined as mono-RIF resistant, isoniazid-susceptible TB. MDR-TB was defined as resistance to both isoniazid and RIF. Pre–XDR-TB was defined as resistance to RIF and isoniazid along with resistance to either one of the fluoroquinolones (ofloxacin, levofloxacin, or moxifloxacin) or second-line injectables (AMK, capreomycin, or kanamycin). XDR-TB was defined as MDR-TB, plus any fluoroquinolone, plus at least one of the drugs bedaquiline and linezolid. Poly–DR-TB was defined as resistance to multiple drugs but not meet to definition of HR-TB, RR-TB, MDR-TB, pre-XDR-TB and XDR-TB.

### Genomic DNA extraction

The cetyltrimethylammonium bromide (CTAB) method was used for DNA extraction:

1) 2% CTAB extraction buffer was preheated in a 65°C water bath;

2) take a small amount of experimental material (about 300 mg) in a mortar and grind it to powder with liquid nitrogen;

3) add 700 μl of 2% CTAB extraction buffer and gently agitate;

4) the grinding liquid is divided into 1.5 ml sterilization centrifuge tube, the height of the grinding liquid accounts for about two-thirds of the tube;

5) place in a water bath or incubator at 65°C, gently shake every 10 min, and remove it after 30–60 min;

6) after cooling for 2 min, add chloroform–isoamyl alcohol (24:1) to the full tube, and violently shake for 2–3 min (if the total genome is extracted, it cannot be violently shaken), so that the two mix evenly;

7) centrifuge at 11,100*g* for 10 min, at the same time, add 600 μl of isopropyl alcohol into another new sterilization centrifuge tube;

8) after centrifugation at 11,100*g* for 1 min, the pipette gently absorbed the clear night and transferred it into the centrifuge tube containing isopropyl alcohol, the centrifuge tube was slowly shaken up and down for 30 s, so that isopropyl alcohol and water layer were fully mixed until DNA flocculent was visible;

9) after centrifugation at 11,100*g* for 1 min, immediately pour out the liquid, be careful not to pour out the white DNA precipitate, and stand the centrifuge tube upside down on the spread paper towel;

10) after 60 s, centrifuge the tube upright, add 720 μl of 75% ethanol and 80 μl of 5 M of sodium acetate, turn it gently, and flip the tip of the tube with your fingers, so that the precipitate and DNA clumps at the bottom of the tube float in the liquid;

11) place it for 30 min to dissolve the impurity of the DNA block;

12) centrifuge at 11,100*g* for 1 min, pour off the liquid, add 800 μl 75% ethanol, and wash the DNA for another 30 min;

13) after centrifugation at 11,100*g* for 30 s, immediately pour out the liquid and stand the centrifuge tube upside down on the spread paper towel; after a few minutes, upright centrifuge tube, dry DNA (natural air dry or dry with air dryer);

14) add 50 μl 0.5 × TE (including RNase) buffer to dissolve DNA, and place it in a 37°C incubator for about 15 h to dissolve RNA.

### WGS and bioinformatics analyses

DNA libraries were constructed using Illumina kits. The average sequencing depth was 124.8, and the coverage rate was 99.3%. Double-ended 150-bp sequencing was performed on an Illumina NovaSeq 6000 platform (Jajou et al, 2019; Peker et al, 2021). SAMTools (v1.6) (Li et al, 2009) and VarScan (v2.3.9) (Koboldt et al, 2009) were used to detect single-nucleic acid polymorphism (SNP) by comparing Bowtie 2 with reference genome H37Rv (GenBank accession number, NC_000962.3). Low-quality SNPS (shred score *Q* < 20 and read depth <5) and locus deletion >10% of the isolates were deleted. SNPS in duplicate regions, PE/PPE genes, and resistance-related genes were excluded from further phylogenetic analysis, and maximum likelihood (M-L) phylogenetic trees were constructed. We compared pairwise genomic distances and defined genomic clusters as genetic distances of strains with no more than 12 SNPS.

WGS was used to detect mutations in the genes that confer resistance to anti-TB drugs including RIF, INH, PZA, EMB, SM, fluoroquinolones (FQs), aminoglycosides, ETO, PAS, cycloserine (Cs), linezolid, bedaquiline, clofazimine, delamanid.

### Statistical analysis

All data were presented as mean ± SD or frequency. Statistical analysis for possible significant association between the different characteristics and different genotypes was performed using Chi-square test. BioNumerics (version 5.0; Applied Maths) was used to construct the minimal spanning trees based on WGS data. A dendrogram was constructed based on the unweighted-pair group method using average linkages and the software package MEGA (version 6.0). The univariate analysis was used to compare differences in different characteristics between the different lineages and clusters. All characteristics underwent multivariate logistic regression analysis. *P*-value of < 0.05 was considered statistical significant.

### Ethics statement

This was retrospective study. Our study was approved by the Committee for Medical Ethics of the Chongqing Public Health Medical Center and Tianjin First Central Hospital.

## Supplementary Material

Reviewer comments
